# The Occurrence and Genomic Characteristics of *mcr-1*-Harboring *Salmonella* from Retail Meats and Eggs in Qingdao, China

**DOI:** 10.3390/foods11233854

**Published:** 2022-11-29

**Authors:** Changan Li, Xiulei Gu, Liping Zhang, Yuqing Liu, Yan Li, Ming Zou, Baotao Liu

**Affiliations:** 1 College of Veterinary Medicine, Qingdao Agricultural University, Qingdao 266109, China; 2 Institute of Animal Science and Veterinary Medicine, Shandong Academy of Agricultural Sciences, Jinan 250100, China; 3 Qingdao Center for Animal Disease Control and Prevention, Qingdao 266000, China

**Keywords:** *Salmonella*, *mcr-1*, plasmids, retail meats and eggs, China

## Abstract

*Salmonella* are widely distributed foodborne pathogens and are often associated with food animal products. Colistin resistance mediated by *mcr-1* is an increasing threat; however, data on the characteristics of *mcr-1*-harboring *Salmonella* among retail foods are still lacking. In this study, retail meats from 24 supermarkets and eggs from nine markets in Qingdao city were investigated to determine the presence and genomic characteristics of *mcr-1*-harboring *Salmonella*. We found the retail meats and eggs were highly contaminated by *Salmonella*, with detection rates of 17.5% (31/177) and 12.3% (16/130), respectively. A total of 76 *Salmonella* isolates were obtained in this study, and 77.6% showed multidrug resistance (MDR). The MDR proportion of egg isolates (97.5%) was significantly higher than that in meat isolates (55.6%) (*p* < 0.05). The most prevalent *Salmonella* serotypes were Typhimurium (56.6%) and Enteritidis (17.1%). Of the 76 *Salmonella* isolates, 40 possessed *mcr-1*. All 40 *mcr-1*-positive isolates were ST34 *S*. Typhimurium and were from eggs of eight brands. Different *mcr-1*-harboring isolates existed in the same egg, and some isolates from different egg samples or brands showed clonal relationships. The *mcr-1* was located on similar IncHI2/HI2A MDR non-conjugative plasmids lacking transfer region, resulting in the failure of conjugation. The phylogenetic tree using genome sequences showed that the *mcr-1*-positive isolates from eggs clustered together with *mcr-1*-positive isolates from chicken and humans in China, revealing that *mcr-1*-positive egg-borne *Salmonella* might be derived from chicken and could potentially trigger outbreaks in humans. The high occurrence of *mcr-1*-harboring *Salmonella* in fresh eggs is alarming, and there is an urgent need to monitor *mcr-1*-harboring *Salmonella* in retail meats and eggs. We report for the first time the role of retail eggs in disseminating *mcr-1*-positive *Salmonella* and the risk of transmission of these MDR pathogens from retail food to humans should be evaluated comprehensively.

## 1. Introduction

Colistin is widely and increasingly used as a last-resort antibiotic for treating severe infections caused by multidrug-resistant (MDR) Gram-negative bacteria, especially carbapenemase producers [1]. However, colistin use in animals has promoted the emergence of a mobilized colistin resistance (*mcr*) gene, *mcr-1*, challenging the efficacy and clinical usefulness of this drug [2]. To date, several subtypes of *mcr* (*mcr-1* to *mcr-10*) have been identified in various Enterobacteriaceae members [1,3,4]. The *mcr-1* gene shows the highest prevalence among members of Enterobacteriaceae, especially *Escherichia coli*, and has been found in samples of different origins around the world in recent years, including humans [5], food-producing animals [6], aquaculture [7], migratory birds [8], wild animals [9], environments [10], flies [11], vegetables [12] and retail meats [13]. 

*Salmonella*, responsible for 180 million diarrheal illnesses that occur globally each year, are widely distributed foodborne pathogens and cause about 298,000 deaths worldwide each year [14]. In Europe, *Salmonella* cause 88,715 confirmed cases of salmonellosis, with 23.4 cases per million people [15]. In the United States, about 1.35 million infections, 26,500 hospitalizations and 420 deaths are caused by *Salmonella* every year (https://www.cdc.gov/salmonella/, accessed on 26 November 2022). In China, it has been also reported that *Salmonella* are responsible for about 70~80% of foodborne pathogenic outbreaks and cause nontyphoidal salmonellosis with an incidence of 626.5 infections per 100,000 persons [16]. To date, more than 2600 serotypes of *Salmonella* have been identified; however, human nontyphoidal salmonellosis are mainly caused by only a few serotypes, especially by *S*. Typhimurium and *S*. Enteritidis [17], which can also cause systemic disease in a wide range of host animals [15]. Foodborne salmonellosis in humans is mainly attributed to *Salmonella* transmission from food animals to humans via the food chain. In Europe, 42.4% of human salmonellosis cases were caused by the eggs of laying hens [15]. The rising incidence of MDR *Salmonella* in food animal products has been a great threat to humans. 

Although the detection rate of *mcr-1* in clinical *Salmonella* isolates in many countries remains very low [18,19], *mcr-1*-positive *Salmonella* isolates have already occurred in food animal products, especially retail meats [20,21], and *S*. Typhimurium has been the prevalent serotype for both clinical and foodborne *mcr-1*-positive *Salmonella* isolates [22,23]. As antimicrobial resistance (AMR) in retail foods has not been brought in line with the national AMR surveillance programmes in China [24], the understanding of *mcr-1*-positive foodborne bacteria is limited. Reports about foodborne bacteria carrying *mcr-1* in China have mainly focused on *E. coli* from retail meats [25,26,27]. Recently, scattered *mcr-1*-positive *Salmonella* isolates have been reported in retail meats in China [28,29]. In contrast, little attention has been paid to retail eggs, which account for a considerable proportion of the human daily diet, although one *S*. Typhimurium recovered from egg has been found to carry *mcr-1* recently [23]. There is an urgent need to investigate the presence and characteristics of *Salmonella* harboring *mcr-1* in food animal products, especially retail eggs.

To increase our understanding of the molecular genetic mechanisms involved in the emergence and dissemination of *mcr-1*-harboring *Salmonella* in retail food, we conducted a surveillance of *mcr-1*-positive *Salmonella* in retail meats and eggs in China and identified the genomic characteristics of prevalent isolates carrying *mcr-1*. Findings of current work should lead to insights for developing effective strategies for controlling *mcr-1*-positive *Salmonella* in retail food and reducing untreatable infections in humans.

## 2. Materials and Methods

### 2.1. Isolation and Serotyping of Salmonella

For this study, 177 retail raw meat samples (31 pork samples and 146 chicken meat samples) belonging to 15 brands were collected from 24 large supermarkets distributed across Qingdao city, China. These 177 unpackaged meat samples included 19 in the year 2017, 49 in 2018 and 109 in 2021. A total of 130 fresh eggs were purchased from four large supermarkets (64 eggs) and five small markets (66 eggs) in Qingdao in 2021. These eggs came from 22 farms about which further information was unfortunately not available. All samples were stored below 8 °C and processed within 24 h after sampling.

Raw meat samples were aseptically homogenized individually, and 25 g of each homogenized meat sample was mixed with 225 mL buffered peptone water (BPW) in stomacher bag. For detecting *Salmonella* within egg, the egg content was obtained according to a previous method [30]. Briefly, we removed adherent materials on the shell surface and then soaked eggs in 70% ethyl alcohol for at least 10 s. The shell was cracked, and a sterile thumb forceps was used to aseptically separate shell from the interior content to generate a hole. The whole egg content including albumen and yolk was poured into sterile bag and then mixed thoroughly. Each homogenized egg content (25 mL) was also mixed with 225 mL BPW and then incubated at 37 °C overnight as the retail meat samples. *Salmonella* isolates were obtained according to the procedures established by China national food safety standard GB 4789.4-2016 [31]. As different *Salmonella* colonies might exist in the same sample, one to three suspected colonies were selected from each sample. The obtained isolates were identified using a *Salmonella*-specific *invA* gene PCR [32]. Thirty-one different serotypes representing the most common clinical *Salmonella* were identified in this study. The serotypes Enteritidis, Typhimurium, Dublin and Pullorum/Gallinarum were identified by PCR as previously described [33,34]. The remaining 27 different serotypes were determined using another method [35]. The PCR amplicons were randomly selected for sequencing to confirmed the serotypes.

### 2.2. Antimicrobial Susceptibility Testing and Identification of Resistance Genes

The minimum inhibitory concentrations (MICs) of ampicillin, ceftiofur, cefotaxime, meropenem, nalidixic acid, enrofloxacin, ciprofloxacin, levofloxacin, tetracycline, doxycycline, tigecycline, streptomycin, kanamycin, gentamicin, amikacin, colistin and fosfomycin were determined by the agar dilution method, and the results (except for tigecycline) were analyzed according to the CLSI criteria [36]. The MIC method used for colistin and resistant breakpoints for colistin and tigecycline were recommended according to a 2019 EUCAST procedure [37]. The presence of *mcr* genes (*mcr-1* to *mcr-10*) was determined by PCRs [3,4], and carbapenemase-encoding genes were also screened as previously described [38]. Plasmid-mediated quinolone resistance (PMQR) and *bla*_CTX-M_ genes were analyzed as we reported previously [39]. 16S rRNA methyltransferases genes were also analyzed [40].

### 2.3. Pulsed-Field Gel Electrophoresis and Conjugation Experiments

Pulsed-field gel electrophoresis (PFGE) was performed to determine the genetic relatedness among the obtained Salmonella isolates [41], and PFGE patterns were analyzed with the BioNumerics software. The multilocus sequence types (MLSTs) of Salmonella carrying mcr genes were also analyzed (https://pubmlst.org/bigsdb?db=pubmlst_salmonella_seqdef, accessed on 26 November 2022). Conjugation of all mcr-carrying isolates was performed using the broth mating method with streptomycin-resistant E. coli C600 as the recipient.

### 2.4. Whole Genome Sequencing and Phylogenetic Analysis 

Because three (S34L1, S34L2 and S34L3), two (S37L1 and S37L3) and two (S58L1 and S58L2) *mcr-1*-positive isolates with different PFGE patterns were recovered from egg sample S34, S37 and S58, respectively; these seven *mcr-1*-harboring isolates were further analyzed using whole-genome sequencing (WGS). Three *mcr-1*-positive isolates (S46L1 from brand 5, S47L1 from brand 5 and S54L1 from brand 6) with identical PFGE patterns from different egg samples/brands were also subjected to WGS. Briefly, total genomic DNA of *mcr-1*-harboring isolates were prepared using the TIANamp Bacteria DNA Kit (Tiangen, Beijing, China). Paired-end sequencing (2 × 150 bp) of genomic DNA was performed using the Illumina HiSeq platform (Illumina, San Diego, CA, USA) to generate enough data sets (≥100 × coverage for each sample). In order to obtain high-quality clean reads, raw reads were filtered to remove the adaptor sequences and the low-quality reads. SPAdes v3.8.2 was used to assemble the Illumina clean reads [42]. Isolates S46L1 and S58L2 with different sizes of plasmids were further sequenced using the Oxford Nanopore MinION sequencer platforms. Illumina and MinION reads were assembled de novo using Unicycler v0.4.7 [43]. The Center for Genomic Epidemiology web tools (https://cge.cbs.dtu.dk/services/, accessed on 26 November 2022) were used to analyze resistance genes and plasmid replicon types.

To track the phylogenetic relationships of the *mcr-1*-positive ST34 *S*. Typhimurium isolates from different origins, we used 10 genome sequences of *Salmonella* obtained from eggs in this study and the genome sequences of the few *mcr-1*-positive ST34 *S*. Typhimurium in the NCBI Pathogen database (https://www.ncbi.nlm.nih.gov/pathogens, accessed on 26 November 2022) including seven from humans, one chicken isolate and one egg isolate. SNPs were determined using the web tool CSI Phylogeny 1.4 (https://cge.food.dtu.dk/services/CSIPhylogeny/, accessed on 26 November 2022) and S46L1 possessing the prevalent PFGE pattern was set as reference. The phylogenetic tree was generated using the online tool iTOL v6 (https://itol.embl.de, accessed on 26 November 2022).

### 2.5. Characterization of mcr-1-Bearing Plasmids 

The complete sequences of *mcr-1*-bearing plasmids were obtained from the two nanopore-sequenced genomes, while the plasmid contigs in the remaining Illumina-sequenced isolates were extracted from the whole-genome sequences with Plasmid SPAdes tool (http://spades.bioinf.spbau.ru/plasmidSPAdes/, accessed on 26 November 2022) using default parameters. Functional annotation of the sequenced genomes was performed using the NCBI Prokaryotic Genome Annotation Pipeline (PGAP) server. The alignment comparison of *mcr-1*-bearing plasmids was visualized by the BRIG version 0.95 [44]. The linear alignment of these plasmids was also performed using Easyfig version 2.1 [45].

### 2.6. Nucleotide Sequence Accession Numbers 

The accession numbers of the chromosomes of S46L1 and S58L2 submitted to NCBI are CP091540 and CP091542, respectively, and accession numbers of pMCR_S46L1 and pMCR_S58L2 are CP091541 and CP091543, respectively. The genome sequences of the remaining eight isolates are as following: S34L1 (JAKKDG000000000), S34L2 (JAKKDH000000000), S34L3 (JAKKDI000000000), S37L1 (JAKKDJ000000000), S37L3 (JAKLWT000000000), S47L1 (JAKLWU000000000), S54L1 (JAKLWV000000000) and S58L1 (JAKLWW000000000).

## 3. Results

### 3.1. Detection Rates and Serotypes of Salmonella in Raw Meats and Eggs

**Figure 1:**
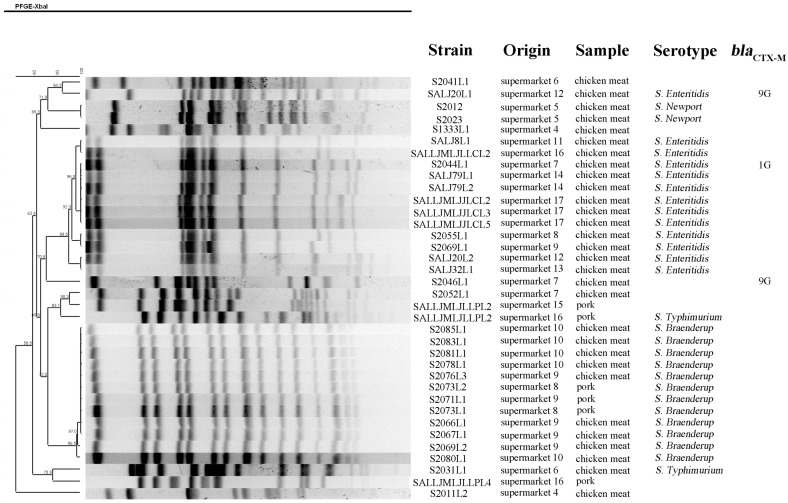
Characteristics and PFGE dendrogram patterns of the 36 *Salmonella* isolates from retail meats of different origins.

**Figure 2:**
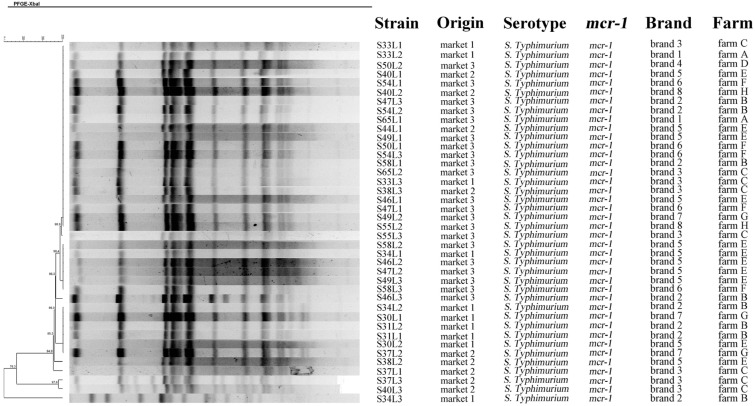
Characteristics and PFGE dendrogram patterns of the 40 *Salmonella* isolates harboring *mcr-1* from retail eggs.

Among the 177 raw meat samples, 36 *Salmonella* isolates were identified in 31 meat samples (17.5%, 31/177) from 14 large supermarkets (Table 1 and Figure 1). Of the 109 meat samples collected in 2021, 21 samples (19.3%) harbored *Salmonella*, and this detection rate was similar with that in samples collected during 2017–2018 (*p* > 0.05). There was no significant difference between the *Salmonella* isolation rate in chicken meats (17.8%, 26/146) and that in pork (16.1%, 5/31) (*p* > 0.05) (Table 1). A total of 16 fresh egg samples of eight brands from three markets harbored *Salmonella*. A group of 40 *Salmonella* isolates were obtained from these 16 egg samples, which came from eight chicken farms (Figure 2). The detection rate of *Salmonella* in eggs (12.3%, 16/130) was similar to that in retail meats (17.5%, 31/177) (*p* > 0.05). Notably, all the *Salmonella*-positive egg samples were collected from small markets, and the detection rate was 24.2% (16/66) (Table 1). 

Here, 70 of the 76 *Salmonella* isolates were successfully serotyped, and *S*. Typhimurium was the most common serotype (56.6%), followed by *S*. Enteritidis (17.1%) and *S*. Braenderup (15.8%) (Table 2). Among the 36 isolates from retail meats, the top two serotypes were *S*. Enteritidis (36.1%, 13/36) and *S*. Braenderup (33.3%, 12/36), while all 40 isolates from fresh eggs belonged to *S*. Typhimurium (Table 2). 

### 3.2. Antimicrobial Resistance Patterns and Genotypes 

The highest resistance rates were found for ampicillin (78.9%) and nalidixic acid (78.9%). Resistance rates to streptomycin, gentamicin, kanamycin and colistin were all above 50.0% (Table 3). No isolate was found to be resistant to meropenem and tigecycline. Notably, the resistance rate to the six antimicrobials (gentamicin, kanamycin, colistin, streptomycin, ampicillin and nalidixic acid) among isolates from eggs was above 90.0% and was significantly higher than that from retail meats (*p* < 0.05) (Table 3). In particular, 59 of the 76 *Salmonella* isolates (77.6%) exhibited MDR, resistant to at least one agent in three or more antimicrobial classes (Table 3). Notably, 97.5% of the 40 *Salmonella* isolates from eggs were MDR and the resistance rate to at least three and four antimicrobial classes in fresh egg isolates was significantly higher than that in isolates from retail meats, respectively (*p* < 0.05) (Table 3). The resistance pattern (ampicillin-colistin-streptomycin-kanamycin-gentamicin-nalidixic acid) was observed in 28 egg-sourced isolates and was also the predominant MDR pattern among the 59 foodborne MDR isolates.

Among the 76 *Salmonella* isolates, three from chicken meats possessed *bla*_CTX-M_ genes (Figure 1). A group of 40 isolates (52.6%) carried *mcr-1*, and all 40 isolates were *S*. Typhimurium. Notably, all 40 *mcr-1*-positive *S*. Typhimurium were recovered from eggs of eight brands, and each of these eight brands came from one chicken farm (Figure 2). Genes *mcr-2*~*mcr-10* were not found in our study. Only one isolate harbored the PMQR gene *oqxAB*, and other PMQR genes were not found in our study. Carbapenemase-encoding genes and 16S rRNA methyltransferases genes were also not identified in our study.

### 3.3. Conjugation and PFGE Analysis

We determined the transferability of *mcr-1* using conjugation three times, but *mcr-1* in all 40 *S*. Typhimurium isolates could not be transferred to *E. coli* C600. All 76 *Salmonella* isolates were successfully subtyped by *Xba*I-PFGE. For the 36 isolates from retail meats, 18 PFGE patterns were identified, and isolates of the same serotype were clustered together (Figure 1). All the *S*. Braenderup isolates had high genetic similarity (>96.5%), and isolates from different types of meat or markets (i.e., isolates S2076L3, S2078L1, S2073L1 and S2071L1) also shared identical PFGE pattern. Clonal relationships were also found among *S*. Enteritidis from different samples within market or different markets (i.e., isolates S2044L1, SALJ79L1 and SALLJMLJJLCL2). Notably, *Salmonella* isolates from the same meat sample had different PFGE patterns (i.e., SALJ20L1 and SALJ20L2) or serotypes (i.e., S2069L1 and S2069L2) (Figure 1). 

All the 40 *S*. Typhimurium isolates harboring *mcr-1* from fresh eggs were ST34 type and most isolates were clustered together (Figure 2). Isolates possessing different PFGE patterns were also identified in the same samples (i.e., isolates S34L1, S34L2 and S34L3 from sample S34; isolates S37L1 and S37L3 from sample S37; isolates S40L1 and S40L3 from sample S40). Clonal relationship was found among the *S*. Typhimurium isolates from different samples of the same brand (i.e., isolates S46L1 and S47L1 from egg brand 5; isolates S37L1, S38L2 and S40L1 from egg brand 3). Notably, *S*. Typhimurium isolates harboring *mcr-1* from eggs of different brands (i.e., S30L1, S31L1, S40L1, S44L1, S47L1, S54L1, S58L1 and S65L2 from egg samples of eight brands) were also found to share identical PFGE pattern.

### 3.4. Genomic Characteristics of mcr-1-Positive S. Typhimurium Isolates 

For each of the 10 sequenced isolates, the number of obtained contigs ranged from 50 to 56, and the contig N50 size was between 318,715 bp and 377,001 bp. All 10 sequenced isolates possessed multiple antimicrobial resistance genes, including resistance to colistin (*mcr-1*), aminoglycosides (*aph(4)-Ia*, *aac(3)-IV*, *aph(6)-Id*, *aac(6′)-Iaa*, and *aph(3″)-Ib*), β-lactams (*bla*_OXA-1_ and *bla*_TEM-1B_), fluoroquinolones (*aac(6′)-Ib-cr* and *gyrA* (D87Y)), phenicols (*catB3*), rifamycin (*arr-3*) and sulfonamides (*sul1* and *sul2*) (Table 4). Isolate S58L2 also carried eight additional resistance genes (*aph(3′)-Ia*, *aadA1*, *aadA2b, sul3, oqxAB, floR, dfrA12,* and *cmlA1*). All 10 *mcr-1*-positive isolates only carried IncHI2/HI2A type plasmid (Table 4).

**Figure 3:**
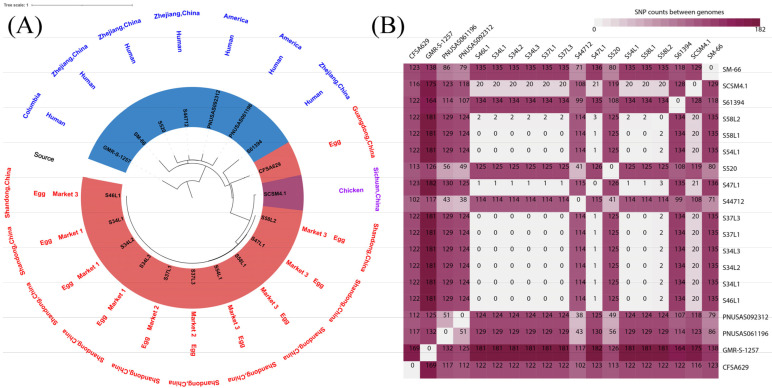
Phylogenetic analysis (**A**) and SNPs variation (**B**) of the 19 *mcr-1*-harboring ST34 *S*. Typhimurium isolates from different countries and different sources, including nine genomes from the NCBI database and 10 isolates in this study.

The genomic phylogeny analysis showed that 182 SNPs were obtained among the 19 *mcr-1*-positive ST34 *S*. Typhimurium isolates, which included isolates from humans and chicken in the NCBI pathogen database. All 10 ST34 *S*. Typhimurium isolates from eggs in our study were clustered together and had a limited number of variations (0 to 2 SNPs), showing a close genetic relationship among these isolates even for those from different samples or brands (Figure 3). Notably, all 10 egg-sourced isolates were also clustered together with the chicken-source isolate SCSM4.1 (NZ_CP047115) in China, with a limited number of variations (20 to 21 SNPs), suggesting chicken feces as the likely contamination source of *mcr-1*-harboring *S*. Typhimurium in eggs (Figure 3). Most human isolates from China and America showed less than 135 SNPs away from our egg-sourced isolates (Figure 3).

### 3.5. Complete Sequence Analysis of mcr-1-Carrying Plasmids 

**Figure 4:**
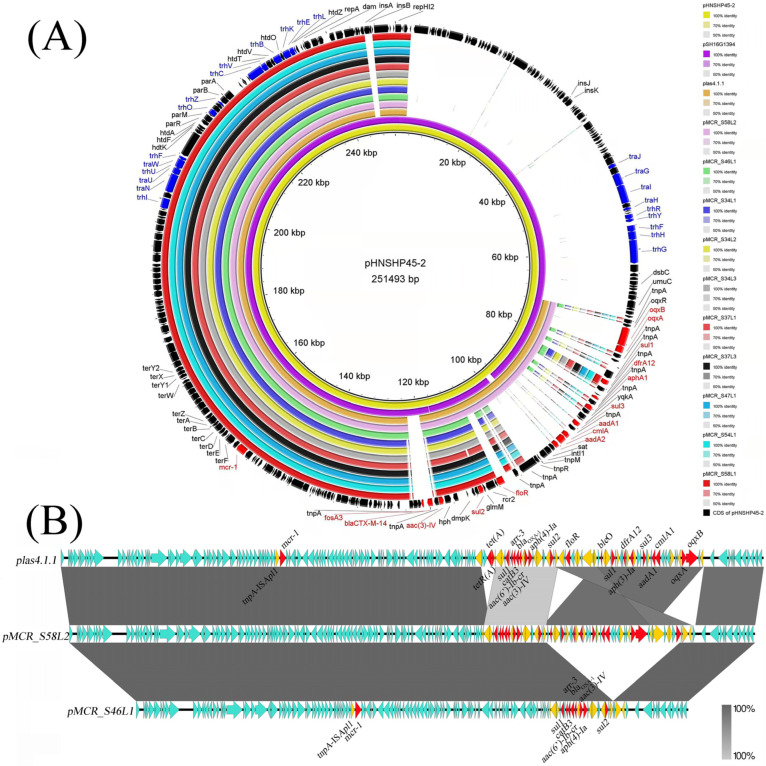
Circular (**A**) and linear (**B**) sequence alignment of *mcr-1*-bearing IncHI2 plasmids in this study and other similar plasmids available from the NCBI database. (**A**) Circular alignment of the nine pMCR_S46L1-like plasmids, pMCR_S58L2 and similar plasmids available from the NCBI database including pHNSHP45-2 (KU341381), pSH16G1394 (NZ_MK477614) and plas4.1.1 (NZ_CP047116). Plasmid pHNSHP45-2 was included as a reference and the outermost ring showed annotations of pHNSHP45-2. The resistance genes and transfer-related genes are marked with red and purple, respectively. (**B**) Linear alignment of *mcr-1*-harboring pMCR_S46L1 (CP091541), pMCR_S58L2 (CP091543) and plas4.1.1 (NZ_CP047116). Insertion sequences or *tnpA* in insertion sequences are highlighted in orange arrows, while resistance genes are indicated by red arrows.

The complete sequences of *mcr-1*-carrying IncHI2/HI2A plasmids pMCR_S58L2 (CP091543, 185529 bp) and pMCR_S46L1 (CP091541, 149407 bp) were obtained. The plasmid contigs in the remaining eight isolates were also identified, and all these isolates also carried only one IncHI2/HI2A type plasmid harboring *mcr-1*. All plasmids in the remaining eight isolates were highly identical to pMCR_S46L1 (Figure 4A). Notably, pMCR_S58L2 covered all sequences of pMCR_S46L1 with an identity of 100.0%; however, pMCR_S46L1 possessed 85% sequences of pMCR_S58L2 (Figure 4). All nine pMCR_S46L1-like plasmids carried nine resistance genes including *mcr-1*, *aph(4)-Ia*, *aac(3)-IV*, *bla*_OXA-1_, *aac(6′)-Ib-cr*, *catB3*, *arr-3*, *sul1* and *sul2* (Figure 4B). In addition to the above nine resistance genes, pMCR_S58L2 harbored nine additional resistance genes including aminoglycosides (*aph(3′)-Ia*, *aadA1* and *aadA2b*), sulfonamides (*sul1* and *sul3*), fluoroquinolones (*oqxAB*), phenicols (*floR* and *cmlA1*) and trimethoprim (*dfrA12*) resistance genes (Figure 4B). In both types of *mcr-1* plasmids, all resistance genes except *mcr-1* were located in the MDR region, and transposase genes within insertion sequences, especially *tnpA*-IS*26*, were adjacent to each of these resistance genes. An MDR region of ~36 kb carrying nine additional resistance genes mentioned above was integrated into the MDR region of pMCR_S46L1, forming the plasmid pMCR_S58L2 (Figure 4B). Only one copy of IS*Apl1* was near *mcr-1* in all *mcr-1*-bearing plasmids in this study, and a heavy metal resistance region possessing multiple tellurium resistance genes (*terYXWZABCDEF*) was also found in these plasmids (Figure 4A). 

In addition to MDR regions, all 10 plasmids also had other backbone structures typical of IncHI2 plasmid pHNSHP45-2 (KU341381), such as plasmid replication (repHIA and repHI2), maintenance system (such as *parA* and *parB*) and transfer-related (*tra* and *trh* series genes) regions (Figure 4A). The 10 plasmids were similar (≥99.99%) to the two typical *mcr-1*-bearing plasmids pHNSHP45-2 (KU341381, 251493 bp) and pSH16G1394 (NZ_MK477614, 251867 bp) from *E. coli* in a pig and *Salmonella* from a diarrheal patient in China, respectively; however, the nine pMCR_S46L1-like plasmids and pMCR_S58L2 possessed only 59% and 70% sequences of the two typical IncHI2 plasmids, respectively (Figure 4A). The typical IncHI2 plasmids (pHNSHP45-2 and pSH16G1394) contained two transfer regions: region 1 including genes *traJ*-*traH* and genes *trhR*-*trhG*; region 2 including *traNUW* and *trhIUFOZCVBKEL* (Figure 4A). We did not identify conjugative transfer region 1 in our 10 plasmids (Figure 4A), accounting for the failure of conjugation. Notably, pMCR_S58L2 was highly similar (100.0% identity and 98.0% coverage) to the *mcr-1*-bearing plasmid plas4.1.1 (NZ_CP047116, 190174 bp) in *Salmonella* from chicken feces in China (Figure 4A), and only a deletion and inverted insertion of small fragments mediated by transposases were found in plasmid plas4.1.1 (Figure 4B). This result suggests that *mcr-1*-harboring plasmids in *S*. Typhimurium in fresh eggs might be derived from plasmids in isolates from chicken feces. 

## 4. Discussion

*Salmonella* are the leading cause of bacterial food poisoning worldwide [14,29], and the antimicrobial resistance of such foodborne pathogens is spreading in many countries [46]. However, data on the characteristics and transmission of foodborne *Salmonella* harboring *mcr-1* are still lacking. In this study, we investigated the detection rate of *Salmonella* among retail food sold in Qingdao, China, and found that retail meats and eggs were widely contaminated by *Salmonella*. Moreover, colistin resistance gene *mcr-1* was frequently found in *Salmonella* isolates from retail food, especially from eggs. In this context, retail food animal products were important reservoirs in spreading *Salmonella* carrying *mcr-1*.

The detection rate of *Salmonella* in retail meats in this study was 17.5%, which was higher than that reported in Hubei Province, China (10.5%) [47], and that reported in the United States (8.1%) [48] but lower than that reported in Hebei Province, China (59.5%) [49]. The detection rate of *Salmonella* in egg samples (12.3%, 16/130) in this study was similar to that in retail eggs in Nigeria (10.0%) [30] but higher than that reported in eggs in southern Brazil (1.25%) [50]. Notably, the detection rate of 10% in the Nigerian study was artificially inflated because the samples were pooled (each sample from five eggs), unlike in this study, where an individual egg was used as one sample. Actually, no *Salmonella* was obtained in market-sourced egg contents, and all the market-sourced *Salmonella* isolates were recovered from eggshells in the previous Nigerian study [30]. The high detection rate of *Salmonella* in egg contents in this study was somewhat unexpected, because retail eggs were paid little attention in previous surveillance studies in China [49]. Notably, all the *Salmonella*-positive egg samples were collected from small markets, which might be because the biosecurity and hygiene measures in the farm suppliers of eggs between small markets and large supermarkets were different. *S*. Typhimurium was the most common serotype in this study, consistent with a previous study in China [23], while it differed from other reports in which the dominant serotype was *S*. Thompson in Iran [51], *S*. Thompson in Hubei Province, China [47] and *S*. Derby in Hebei Province, China [49]. The differences in the dominant serotypes among studies might be the result of geographical differences and different sample types. Thus, ongoing surveillance is necessary to monitor the prevalence of *Salmonella* in various sample types and areas. 

The high resistance rates to ampicillin and streptomycin in *Salmonella* from retail food observed in this study agree with the fact that these antimicrobials are widely used in food-producing animals [52]. The observed high proportion of MDR isolates was up to 77.6%, and the MDR proportion in fresh egg isolates (97.5%) was significantly higher than that in retail meat isolates (55.6%) suggesting that in addition to retail meats, fresh eggs might have been underestimated in spreading antimicrobial resistance. As colistin has been a last-resort antimicrobial for defending against MDR Gram-negative bacteria, we are surprised to see the high detection rate of *mcr-1* in *Salmonella* from food products. Given *mcr-1* has been found in *Salmonella* from animals in many countries [6,53] and also occurred in *Salmonella* of human origin [17,19], food products, especially fresh eggs, may play a key role in disseminating such pathogens between animals and humans. In this study, all the *mcr-1*-positive *Salmonella* isolates were ST34 *S*. Typhimurium, similar to the finding that the majority of *mcr-1*-positive *Salmonella* isolates in humans and animals were ST34 *S*. Typhimurium [54,55]. Because ST34 *S*. Typhimurium has been reported across European countries, Japan and China [56,57], the relationship between ST34 *S*. Typhimurium and *mcr-1* needs to be further elucidated, and international coordinated intervention strategies are required to limit the further dissemination of *mcr-1*-harboring ST34 *S*. Typhimurium around the world.

All 40 *S*. Typhimurium isolates from eggs harbored *mcr-1* gene and *mcr-1*-positve isolates with different PFGE patterns were also identified in the same eggs, indicating that fresh eggs were important reservoirs of MCR-1-producers and that the traditional method of one isolate from each sample would underestimate the detection rate of *mcr-1*-positve *Salmonella* by missing out such isolates. Clonal relationships were found among the *mcr-1*-positive *S*. Typhimurium isolates from different egg samples of the same or different chicken farms. This phenomenon could be explained as that clonal spread of *mcr-1*-positive *Salmonella* exists among and within chicken farms, as the contamination of *mcr-1*-positive *Salmonella* in eggs might be derived from chicken. The source of such resistant pathogens in retail food products should be traced in the future. 

The *mcr-1* gene has been reported in plasmids with different replicon types including IncI2, IncX4, IncHI2, IncHI1, IncP, IncFII and IncFIB [1,17], and the primary replicon type varies among different Enterobacteriaceae members. For instance, IncI2 and IncX4 were the two primary replicon types of plasmids harboring *mcr-1* in *E. coli* from animals [6] and humans [5]. However, *mcr-1*-carrying IncHI2 plasmid was the primary type in *Salmonella* from animals [57] and humans [17]. Most of the IncHI2 plasmids carrying *mcr-1* reported previously were 210~260 kb in size and were also transferable, suggesting that these plasmids harbored complete functional transfer regions. Actually, these typical transferable IncHI2 plasmids contained two transfer regions as previously reported [39]. However, all *mcr-1*-bearing IncHI2 plasmids sequenced in this study were 149 kb or 186 kb and did not possess the conjugative transfer region including genes *traJGIH* and *trhRYXFHG*, accounting for the failure of the transferability of these plasmids by conjugation. In addition to *mcr-1*, all the IncHI2 plasmids observed in this study also encoded resistance genes to aminoglycosides (*aac(3)-IV*, *aph(4)-Ia*), quinolones (*aac(6’)-Ib-cr*) and sulphonamides (*sul1*, *sul2*), which were all relevant drugs for both humans and animals. In order to limit the further spread of *mcr-1*, the adverse effects of these non-polymyxin antimicrobial agents on the spread of colistin resistance in both animals and humans deserve more attention.

The *mcr-1* has been often found in various combinations with one or two copies of IS*Apl1* or devoid of IS element, and IS elements especially the IS*Apl1* have made an important contribution to the rapid dissemination of *mcr-1* [1]. In this study, one copy of IS*Apl1*, rather than IS*Apl1*-flanked composite transposon (Tn*6330*), was near *mcr-1* in all our observed *mcr-1*-bearing plasmids. It is reported that heavy metals including tellurium in environments may contribute to the co-selection of resistance genes such as *mcr-1* in clinically important pathogens, and this contribution can be attributed to the relevant heavy metal resistance-encoding genes [58]. The *mcr-1*-bearing plasmids in this study possessed multiple tellurium resistance genes (*terYXWZABCDEF*), further facilitating the spread of *mcr-1*-bearing pathogens.

All the *mcr-1*-positive ST34 *S*. Typhimurium isolates from different egg samples or brands in our study had a limited number of variations (0 to 2 SNPs), providing evidence of a clonal relationship among these isolates. The rapid expansion of colistin-resistant *Salmonella* in retail eggs due to clonal spread presents a potential public health threat. This threat is proven by the observation of few SNP differences between isolates from retail eggs tested in this study and humans in China [22], suggesting a likely epidemiological association between fresh eggs and human cases. A close relationship was also found between the *mcr-1*-bearing *S*. Typhimurium isolates from eggs and chicken. The similar *mcr-1*-carrying plasmid as those in this study was also identified in *S*. Typhimurium SCSM4.1 (NZ_CP047115) from chicken in China. These results suggest that chicken is the likely contamination source of *mcr-1*-bearing *S*. Typhimurium in eggs. 

## 5. Conclusions

We reported a high detection rate of *Salmonella* in food animal products at retail markets in Qingdao, China. Retail eggs carried *mcr-1*-bearing ST34 *S*. Typhimurium frequently and might have been underestimated vehicles of *Salmonella* in spreading antimicrobial resistance including colistin resistance. Resistance gene *mcr-1* was located on similar IncHI2 MDR non-conjugative plasmids lacking transfer region and the *mcr-1*-positive *S*. Typhimurium isolates from different egg samples or brands showed clonal relationships. A phylogenetic tree based on genome sequences revealed that the *mcr-1*-positive *Salmonella* from eggs in this study might have derived from chicken and could potentially trigger outbreaks in humans. Our findings may help explain the increasing occurrence and transmission of *mcr-1*-harboring *Salmonella* in the community. The high detection rate of *mcr-1*-harboring *Salmonella* in food animal products especially fresh eggs is alarming and constitutes a food safety issue. Sustained surveillance needs be conducted to monitor the presence of *Salmonella* with *mcr-1* in food animal products, and control measures need to be taken to ensure food consumers’ health. To our knowledge, this is the first report of the high detection rate of *mcr-1*-harboring *Salmonella* in fresh eggs.

## Figures and Tables

**Table 1:** Isolation rates of *Salmonella* in different types of retail foods.

Sample Types	Isolation Rates of *Salmonella* (%)	
	Small Markets	Large Supermarkets
Raw meat samples (n = 177)	-	17.5 (31/177)
Chicken meat (n = 146)	-	17.8 (26/146)
Pork (n = 31)	-	16.1 (5/31)
Fresh eggs (n = 130)	24.2 (16/66)	0.0 (0/64)

**Table 2:** Distribution of the serotypes of 76 *Salmonella* isolates in retail food from different sources.

Origins	Ratio of Different Serotypes (%)				
	Enteritidis	Typhimurium	Braenderup	Newport	Not Identified
Meat (n = 36)	36.1 (13/36)	8.3 (3/36)	33.3 (12/36)	5.6 (2/36)	16.7 (6/36)
Chicken meat	43.3 (13/30)	3.3 (1/30)	30.0 (9/30)	6.7 (2/30)	16.7 (5/30)
Pork	0.0 (0/6)	33.3 (2/6)	50.0 (3/6)	0.0 (0/6)	16.7 (1/6)
Fresh eggs (n = 40)	0.0 (0/40)	100.0 (40/40)	0.0 (0/40)	0.0 (0/40)	0.0 (0/40)
Total (n = 76)	17.1 (13/76)	56.6 (43/76)	15.8 (12/76)	2.6 (2/76)	7.9 (6/76)

**Table 3:** Antimicrobial resistance phenotypes of 76 *Salmonella* isolates from different sources.

Antimicrobial Agents	Resistance Rates (%)		
	Retail Meats (n = 36)	Fresh Eggs (n = 40)	Total (n = 76)
β-lactams			
ampicillin	55.6 (20/36) ^a^	100.0 (40/40) ^b^	78.9 (60/76)
ceftiofur	8.3 (3/36) ^a^	2.5 (1/40) ^a^	5.3 (4/76)
cefotaxime	8.3 (3/36) ^a^	0.0 (0/40) ^a^	3.9 (3/76)
meropenem	0.0 (0/36)	0.0 (0/40)	0.0 (0/76)
Quinolones			
nalidixic acid	55.6 (20/36) ^a^	100.0 (40/40) ^b^	78.9 (60/76)
enrofloxacin	8.3 (3/36) ^a^	0.0 (0/40) ^a^	3.9 (3/76)
ciprofloxacin	8.3 (3/36) ^a^	5.0 (2/40) ^a^	6.6 (5/76)
levofloxacin	5.6 (2/36) ^a^	0.0 (0/40) ^a^	2.6 (2/76)
Tetracyclines			
tetracycline	36.1 (13/36) ^a^	0.0 (0/40) ^b^	17.1 (13/76)
doxycycline	36.1 (13/36) ^a^	0.0 (0/40) ^b^	17.1 (13/76)
tigecycline	0.0 (0/36)	0.0 (0/40)	0.0 (0/76)
Aminoglycosides			
streptomycin	44.4 (16/36) ^a^	100.0 (40/40) ^b^	73.7 (56/76)
kanamycin	25.0 (9/36) ^a^	92.5 (37/40) ^b^	60.5 (46/76)
gentamicin	33.3 (12/36) ^a^	97.5 (39/40) ^b^	67.1 (51/76)
amikacin	2.8 (1/36) ^a^	0.0 (0/40) ^a^	1.3 (1/76)
Lipopeptides			
colistin	0.0 (0/36) ^a^	100.0 (40/40) ^b^	52.6 (40/76)
Others			
fosfomycin	8.3 (3/36) ^a^	2.5 (1/40) ^a^	5.3 (4/76)
≥3 (MDR)	55.6 (20/36) ^a^	97.5 (39/40) ^b^	77.6 (59/76)
≥4	27.8 (10/36) ^a^	97.5 (39/40) ^b^	64.5 (49/76)
≥5	5.6 (2/36) ^a^	2.5 (1/40) ^a^	3.9 (3/76)

Different lowercase letters (^a^ and ^b^) stand for significant differences (*p* < 0.05).

**Table 4:** Characteristics of the 10 *mcr-1*-positive *S*. Typhimurium isolates derived from whole genome data analysis.

Isolates	Egg Brands	Markets	Resistance Genes	Replicon Type
S46L1	Brand 5	Market 3	*mcr-1, aph(4)-Ia, aac(6′)-Ib-cr, aac(3)-IV, aph(6)-Id, aac(6′)-Iaa, aph(3″)-Ib, sul1, sul2, arr-3, bla*_OXA-1_*, bla*_TEM-1B_*, catB3, gyrA* (D87Y)	IncHI2/HI2A
S58L2	Brand 7	Market 3	*mcr-1, aph(4)-Ia, aac(6′)-Ib-cr, aac(3)-IV, aph(6)-Id, aac(6′)-Iaa, aph(3″)-Ib, aph(3′)-Ia, aadA1, aadA2b, sul1, sul2, sul3, arr-3, bla*_OXA-1_*, bla*_TEM-1B_*, catB3, gyrA* (D87Y)*, oqxAB, floR, dfrA12, cmlA1*	IncHI2/HI2A
S34L1	Brand 2	Market 1	*mcr-1, aph(4)-Ia, aac(6′)-Ib-cr, aac(3)-IV, aph(6)-Id, aac(6′)-Iaa, aph(3″)-Ib, sul1, sul2, arr-3, bla*_OXA-1_*, bla*_TEM-1B_*, catB3, gyrA* (D87Y)	IncHI2/HI2A
S34L2	Brand 2	Market 1	*mcr-1, aph(4)-Ia, aac(6′)-Ib-cr, aac(3)-IV, aph(6)-Id, aac(6′)-Iaa, aph(3″)-Ib, sul1, sul2, arr-3, bla*_OXA-1_*, bla*_TEM-1B_*, catB3, gyrA* (D87Y)	IncHI2/HI2A
S34L3	Brand 2	Market 1	*mcr-1, aph(4)-Ia, aac(6′)-Ib-cr, aac(3)-IV, aph(6)-Id, aac(6′)-Iaa, aph(3″)-Ib, sul1, sul2, arr-3, bla*_OXA-1_*, bla*_TEM-1B_*, catB3, gyrA* (D87Y)	IncHI2/HI2A
S37L1	Brand 3	Market 2	*mcr-1, aph(4)-Ia, aac(6′)-Ib-cr, aac(3)-IV, aph(6)-Id, aac(6′)-Iaa, aph(3″)-Ib, sul1, sul2, arr-3, bla*_OXA-1_*, bla*_TEM-1B_*, catB3, gyrA* (D87Y)	IncHI2/HI2A
S37L3	Brand 3	Market 2	*mcr-1, aph(4)-Ia, aac(6′)-Ib-cr, aac(3)-IV, aph(6)-Id, aac(6′)-Iaa, aph(3″)-Ib, sul1, sul2, arr-3, bla*_OXA-1_*, bla*_TEM-1B_*, catB3, gyrA* (D87Y)	IncHI2/HI2A
S47L1	Brand 5	Market 3	*mcr-1, aph(4)-Ia, aac(6′)-Ib-cr, aac(3)-IV, aph(6)-Id, aac(6′)-Iaa, aph(3″)-Ib, sul1, sul2, arr-3, bla*_OXA-1_*, bla*_TEM-1B_*, catB3, gyrA* (D87Y)	IncHI2/HI2A
S54L1	Brand 6	Market 3	*mcr-1, aph(4)-Ia, aac(6′)-Ib-cr, aac(3)-IV, aph(6)-Id, aac(6′)-Iaa, aph(3″)-Ib, sul1, sul2, arr-3, bla*_OXA-1_*, bla*_TEM-1B_*, catB3, gyrA* (D87Y)	IncHI2/HI2A
S58L1	Brand 7	Market 3	*mcr-1, aph(4)-Ia, aac(6′)-Ib-cr, aac(3)-IV, aph(6)-Id, aac(6′)-Iaa, aph(3″)-Ib, sul1, sul2, arr-3, bla*_OXA-1_*, bla*_TEM-1B_*, catB3, gyrA* (D87Y)	IncHI2/HI2A

## Data Availability

Data is contained within the article.

## References

[1] Sun J., Zhang H., Liu Y.H., Feng Y. (2018). Towards Understanding MCR-like Colistin Resistance. Trends Microbiol..

[2] Liu Y.Y., Wang Y., Walsh T.R., Yi L.X., Zhang R., Spencer J., Doi Y., Tian G., Dong B., Huang X. (2016). Emergence of Plasmid-Mediated Colistin Resistance Mechanism MCR-1 in Animals and Human Beings in China: A Microbiological and Molecular Biological Study. Lancet Infect. Dis..

[3] Wang C., Feng Y., Liu L., Wei L., Kang M., Zong Z. (2020). Identification of Novel Mobile Colistin Resistance Gene *mcr-10*. Emerg. Microbes Infect..

[4] Borowiak M., Baumann B., Fischer J., Thomas K., Deneke C., Hammerl J.A., Szabo I., Malorny B. (2020). Development of a Novel *mcr-6* to *mcr-9* Multiplex PCR and Assessment of *mcr-1* to *mcr-9* Occurrence in Colistin-Resistant *Salmonella enterica* Isolates From Environment, Feed, Animals and Food (2011–2018) in Germany. Front. Microbiol..

[5] Shen Y., Wu Z., Wang Y., Zhang R., Zhou H.W., Wang S., Lei L., Li M., Cai J., Tyrrell J. (2018). Heterogeneous and Flexible Transmission of *mcr-1* in Hospital-Associated *Escherichia coli*. MBio.

[6] Barbieri N.L., Pimenta R.L., de Melo D.A., Nolan L.K., de Souza M.M.S., Logue C.M. (2021). *mcr-1* Identified in Fecal *Escherichia coli* and Avian Pathogenic *E. coli* (APEC) From Brazil. Front. Microbiol..

[7] Shen Y., Lv Z., Yang L., Liu D., Ou Y., Xu C., Liu W., Yuan D., Hao Y., He J. (2019). Integrated Aquaculture Contributes to the Transfer of *mcr-1* between Animals and Humans via the Aquaculture Supply Chain. Environ. Int..

[8] Zhang Y., Kuang X., Liu J., Sun R.Y., Li X.P., Sun J., Liao X.P., Liu Y.H., Yu Y. (2021). Identification of the Plasmid-Mediated Colistin Resistance Gene *mcr-1* in *Escherichia coli* Isolates From Migratory Birds in Guangdong, China. Front. Microbiol..

[9] Torres R.T., Cunha M.V., Araujo D., Ferreira H., Fonseca C., Palmeira J.D. (2021). Emergence of Colistin Resistance Genes (*mcr-1*) in *Escherichia coli* among Widely Distributed Wild Ungulates. Environ. Pollut..

[10] Teng C.H., Wu P.C., Tang S.L., Chen Y.C., Cheng M.F., Huang P.C., Ko W.C., Wang J.L. (2021). A Large Spatial Survey of Colistin-Resistant Gene *mcr-1*-Carrying *E. coli* in Rivers across Taiwan. Microorganisms.

[11] Yang Q.E., Tansawai U., Andrey D.O., Wang S., Wang Y., Sands K., Kiddee A., Assawatheptawee K., Bunchu N., Hassan B. (2019). Environmental Dissemination of *mcr-1* Positive Enterobacteriaceae by Chrysomya spp. (Common Blowfly): An Increasing Public Health Risk. Environ. Int..

[12] Liu B.T., Li X., Zhang Q., Shan H., Zou M., Song F.J. (2019). Colistin-Resistant *mcr*-Positive Enterobacteriaceae in Fresh Vegetables, an Increasing Infectious Threat in China. Int. J. Antimicrob. Agents.

[13] Kim S., Kim H., Kang H.S., Kim Y., Kim M., Kwak H., Ryu S. (2020). Prevalence and Genetic Characterization of *mcr-1*-Positive *Escherichia coli* Isolated from Retail Meats in South Korea. J. Microbiol. Biotechnol..

[14] Besser J.M. (2018). *Salmonella* Epidemiology: A Whirlwind of Change. Food Microbiol..

[15] Bonardi S. (2017). *Salmonella* in the Pork Production Chain and Its Impact on Human Health in the European Union. Epidemiol. Infect..

[16] Wu B., Ed-Dra A., Pan H., Dong C., Jia C., Yue M. (2021). Genomic Investigation of *Salmonella* Isolates Recovered From a Pig Slaughtering Process in Hangzhou, China. Front. Microbiol..

[17] Lu X., Zeng M., Xu J., Zhou H., Gu B., Li Z., Jin H., Wang X., Zhang W., Hu Y. (2019). Epidemiologic and Genomic Insights on *mcr-1*-Harbouring *Salmonella* from Diarrhoeal Outpatients in Shanghai, China, 2006-2016. EBioMedicine.

[18] Cui M., Zhang J., Gu Z., Li R., Chan E.W., Yan M., Wu C., Xu X., Chen S. (2017). Prevalence and Molecular Characterization of *mcr-1*-Positive *Salmonella* Strains Recovered from Clinical Specimens in China. Antimicrob. Agents Chemother..

[19] Fortini D., Owczarek S., Dionisi A.M., Lucarelli C., Arena S., Carattoli A., Villa L., Garcia-Fernandez A., Enter-Net Italia Colistin Resistance Study Group (2022). Colistin Resistance Mechanisms in Human *Salmonella enterica* Strains Isolated by the National Surveillance Enter-Net Italia (2016–2018). Antibiotics.

[20] Hu Y., Fanning S., Gan X., Liu C., Nguyen S., Wang M., Wang W., Jiang T., Xu J., Li F. (2019). *Salmonella* Harbouring the *mcr-1* Gene Isolated from Food in China between 2012 and 2016. J. Antimicrob. Chemother..

[21] Rau R.B., de Lima-Morales D., Wink P.L., Ribeiro A.R., Martins A.F., Barth A.L. (2018). Emergence of *mcr-1* Producing *Salmonella enterica* Serovar Typhimurium from Retail Meat: First Detection in Brazil. Foodborne Pathog. Dis..

[22] Luo Q., Wan F., Yu X., Zheng B., Chen Y., Gong C., Fu H., Xiao Y., Li L. (2020). MDR *Salmonella enterica* Serovar Typhimurium ST34 Carrying *mcr-1* Isolated from Cases of Bloodstream and Intestinal Infection in Children in China. J. Antimicrob. Chemother..

[23] Hu Y., Fanning S., Nguyen S.V., Wang W., Liu C., Cui X., Dong Y., Gan X., Xu J., Li F. (2021). Emergence of a *Salmonella enterica* Serovar Typhimurium ST34 isolate, CFSA629, Carrying a Novel *mcr-1.19* Variant Cultured from Egg in China. J. Antimicrob. Chemother..

[24] Wu Y.-N., Chen J.-S. (2018). Food Safety Monitoring and Surveillance in China: Past, Present and Future. Food Control.

[25] Liu X., Li R., Zheng Z., Chen K., Xie M., Chan E.W., Geng S., Chen S. (2017). Molecular Characterization of *Escherichia coli* Isolates Carrying *mcr-1*, *fosA3*, and Extended-Spectrum-beta-Lactamase Genes from Food Samples in China. Antimicrob. Agents Chemother..

[26] Tang B., Change J., Luo Y., Jiang H., Liu C., Xiao X., Ji X., Yang H. (2022). Prevalence and Characteristics of the *mcr-1* Gene in Retail Meat Samples in Zhejiang Province, China. J. Microbiol..

[27] Liu X., Geng S., Chan E.W., Chen S. (2019). Increased Prevalence of *Escherichia coli* Strains from Food Carrying *bla*_NDM_ and *mcr-1*-Bearing Plasmids that Structurally Resemble Those of Clinical Strains, China, 2015 to 2017. Eurosurveillance.

[28] Lyu N., Feng Y., Pan Y., Huang H., Liu Y., Xue C., Zhu B., Hu Y. (2021). Genomic Characterization of *Salmonella enterica* Isolates From Retail Meat in Beijing, China. Front. Microbiol..

[29] Hu Y., Nguyen S.V., Wang W., Gan X., Dong Y., Liu C., Cui X., Xu J., Li F., Fanning S. (2021). Antimicrobial Resistance and Genomic Characterization of Two *mcr-1*-Harboring Foodborne *Salmonella* Isolates Recovered in China, 2016. Front. Microbiol..

[30] Agbaje M., Ayo-Ajayi P., Kehinde O., Omoshaba E., Dipeolu M., Fasina F.O. (2021). *Salmonella* Characterization in Poultry Eggs Sold in Farms and Markets in Relation to Handling and Biosecurity Practices in Ogun State, Nigeria. Antibiotics.

[31] (2016). National Food Safety Standard Food Microbiological Examination: Salmonella.

[32] Ferretti R., Mannazzu I., Cocolin L., Comi G., Clementi F. (2001). Twelve-Hour PCR-Based Method for Detection of *Salmonella* spp. in Food. Appl. Environ. Microbiol..

[33] He X., Xu X., Li K., Liu B., Yue T. (2016). Identification of *Salmonella enterica* Typhimurium and Variants Using a Novel Multiplex PCR Assay. Food Control.

[34] Xiong D., Song L., Tao J., Zheng H., Zhou Z., Geng S., Pan Z., Jiao X. (2017). An Efficient Multiplex PCR-Based Assay as a Novel Tool for Accurate Inter-Serovar Discrimination of *Salmonella* Enteritidis, *S*. Pullorum/Gallinarum and *S*. Dublin. Front. Microbiol..

[35] Kim S., Frye J.G., Hu J., Fedorka-Cray P.J., Gautom R., Boyle D.S. (2006). Multiplex PCR-Based Method for Identification of Common Clinical Serotypes of *Salmonella enterica* subsp. enterica. J. Clin. Microbiol..

[36] CLSI (2015). Performance Standards for Antimicrobial Susceptibility Testing; Twenty-Fifth Informational Supplement. CLSI Document M100-S25.

[37] EUCAST (2019). Breakpoint Tables for Interpretation of MICs and Zone Diameters, Version 9.0. Växjö, Sweden: European Committee on Antimicrobial Susceptibility Testing. http://www.eucast.org/clinical_breakpoints.

[38] Poirel L., Walsh T.R., Cuvillier V., Nordmann P. (2011). Multiplex PCR for Detection of Acquired Carbapenemase Genes. Diagn. Microbiol. Infect. Dis..

[39] Liu B.T., Song F.J., Zou M. (2019). Characterization of Highly Prevalent Plasmids Coharboring *mcr-1*, *oqxAB*, and *bla*_CTX-M_ and Plasmids Harboring *oqxAB* and *bla*_CTX-M_ in *Escherichia coli* Isolates from Food-Producing Animals in China. Microb. Drug Resist..

[40] Taylor E., Sriskandan S., Woodford N., Hopkins K.L. (2018). High Prevalence of 16S rRNA Methyltransferases among Carbapenemase-Producing Enterobacteriaceae in the UK and Ireland. Int. J. Antimicrob. Agents.

[41] Gautom R.K. (1997). Rapid Pulsed-Field Gel Electrophoresis Protocol for Typing of *Escherichia coli* O157:H7 and other Gram-Negative Organisms in 1 Day. J. Clin. Microbiol..

[42] Bankevich A., Nurk S., Antipov D., Gurevich A.A., Dvorkin M., Kulikov A.S., Lesin V.M., Nikolenko S.I., Pham S., Prjibelski A.D. (2012). SPAdes: A New Genome Assembly Algorithm and Its Applications to Single-Cell Sequencing. J. Comput. Biol. J. Comput. Mol. Cell Biol..

[43] Wick R.R., Judd L.M., Gorrie C.L., Holt K.E. (2017). Unicycler: Resolving Bacterial Genome Assemblies from Short and Long Sequencing Reads. PLoS Comput. Biol..

[44] Alikhan N.F., Petty N.K., Ben Zakour N.L., Beatson S.A. (2011). BLAST Ring Image Generator (BRIG): Simple Prokaryote Genome Comparisons. BMC Genom..

[45] Sullivan M.J., Petty N.K., Beatson S.A. (2011). Easyfig: A Genome Comparison Visualizer. Bioinformatics.

[46] Parisi A., Crump J.A., Glass K., Howden B.P., Furuya-Kanamori L., Vilkins S., Gray D.J., Kirk M.D. (2018). Health Outcomes from Multidrug-Resistant *Salmonella* Infections in High-Income Countries: A Systematic Review and Meta-Analysis. Foodborne Pathog. Dis..

[47] Zhou M., Li X., Hou W., Wang H., Paoli G.C., Shi X. (2019). Incidence and Characterization of *Salmonella* Isolates From Raw Meat Products Sold at Small Markets in Hubei Province, China. Front. Microbiol..

[48] Yin X., M’Ikanatha N.M., Nyirabahizi E., McDermott P.F., Tate H. (2021). Antimicrobial Resistance in Non-Typhoidal *Salmonella* from Retail Poultry Meat by Antibiotic Usage-Related Production Claims—United States, 2008–2017. Int. J. Food Microbiol..

[49] Wang Z., Zhang J., Liu S., Zhang Y., Chen C., Xu M., Zhu Y., Chen B., Zhou W., Cui S. (2022). Prevalence, Antimicrobial Resistance, and Genotype Diversity of *Salmonella* Isolates Recovered from Retail Meat in Hebei Province, China. Int. J. Food Microbiol..

[50] Haubert L., Maia D.S.V., Rauber Wurfel S.F., Vaniel C., da Silva W.P. (2022). Virulence Genes and Sanitizers Resistance in *Salmonella* Isolates from Eggs in Southern Brazil. J. Food Sci. Technol..

[51] Sodagari H.R., Mashak Z., Ghadimianazar A. (2015). Prevalence and Antimicrobial Resistance of *Salmonella* Serotypes Isolated from Retail Chicken Meat and Giblets in Iran. J. Infect. Dev. Ctries..

[52] Krishnasamy V., Otte J., Silbergeld E. (2015). Antimicrobial Use in Chinese Swine and Broiler Poultry Production. Antimicrob. Resist. Infect. Control.

[53] Elbediwi M., Beibei W., Pan H., Jiang Z., Biswas S., Li Y., Yue M. (2020). Genomic Characterization of *mcr-1*-Carrying *Salmonella enterica* Serovar 4,[5],12:i:- ST 34 Clone Isolated From Pigs in China. Front. Bioeng. Biotechnol..

[54] Zhang H., Xiang Y., Huang Y., Liang B., Xu X., Xie J., Du X., Yang C., Liu H., Liu H. (2021). Genetic Characterization of *mcr-1*-Positive Multidrug-Resistant *Salmonella enterica* Serotype Typhimurium Isolated From Intestinal Infection in Children and Pork Offal in China. Front. Microbiol..

[55] Li X.P., Fang L.X., Song J.Q., Xia J., Huo W., Fang J.T., Liao X.P., Liu Y.H., Feng Y., Sun J. (2016). Clonal Spread of *mcr-1* in PMQR-Carrying ST34 *Salmonella* Isolates from Animals in China. Sci. Rep..

[56] Arai N., Sekizuka T., Tamamura Y., Tanaka K., Barco L., Izumiya H., Kusumoto M., Hinenoya A., Yamasaki S., Iwata T. (2018). Phylogenetic Characterization of *Salmonella enterica* Serovar Typhimurium and Its Monophasic Variant Isolated from Food Animals in Japan Revealed Replacement of Major Epidemic Clones in the Last 4 Decades. J. Clin. Microbiol..

[57] Yi L., Wang J., Gao Y., Liu Y., Doi Y., Wu R., Zeng Z., Liang Z., Liu J.H. (2017). *mcr-1*-Harboring *Salmonella enterica* Serovar Typhimurium Sequence Type 34 in Pigs, China. Emerg. Infect. Dis..

[58] Figueiredo R., Card R.M., Nunez-Garcia J., Mendonca N., da Silva G.J., Anjum M.F. (2019). Multidrug-Resistant *Salmonella enterica* Isolated from Food Animal and Foodstuff May Also Be Less Susceptible to Heavy Metals. Foodborne Pathog. Dis..

